# Local Treatment of the Commoner Acute Throat Affections

**Published:** 1908-05-09

**Authors:** J. W. Miller

**Affiliations:** late Assistant Medical Officer of Health, Blackburn.


### The General Practitioner's Column.

[Contributions to this Column are invited, and if accepted will be paid for.]

### LOCAL TREATMENT OF THE COMMONER ACUTE THROAT AFFECTIONS.

By J. W. MILLER, M.D., D.P.H., late Assistant Medical Officer of Health, Blackburn.

The important point in the local treatment of acute throat affections is the application of a solution which will have a cleansing effect by dissolving the mucus, and in addition an antiseptic action on the mucous membrane of the throat. Where there is much discharge of mucus and pus, as in the severer forms of scarlet fever, quinsy, follicular tonsillitis, and septic throats, simply painting the throat with an antiseptic solution several times daily is inefficient, and more vigorous treatment is necessary. In the case of children, syringing, douching, or swabbing is desirable, and for adults, in addition, gargling, if not too painful.

For syringing, a four-ounce ball syringe or a Higginson's syringe can be used, care being taken that too much force is not used, as there is a danger of fluid being forced along the Eustachian tube into the middle ear, with the attendant results of pain and inflammation; with douching this accident is less likely to happen, as the stream is a gradual one. In the most severe cases, where the constitutional symptoms are marked, swabbing causes less disturbance to the patient. Where there is much inflammation of the mucous membrane of the throat, along with a considerable amount of discharge, the throat may be treated every hour or two hours, and in cases of moderate severity every four hours.

The anti-toxin treatment is chiefly relied upon in cases of diphtheria, and very little local treatment is necessary even in severe cases; syringing may upset the patient too much when the constitutional symptoms are marked and the heart is weak. Swabbing or painting the throat every three or four hours will cause less disturbance than syringing or douching. In regard to the solutions used, sodium chloride, sodium bicarbonate, and borax will be found useful in dissolving mucus; they can be used separately or in combination, one or two drachms of either salt to a pint of warm water, or half a drachm of each salt in combination. The throat may receive a preliminary cleansing with the above solutions and an antiseptic applied, or the two may be used together in one solution.

The choice of antiseptics is limited—many are unsuitable either on account of their taste and smell or irritant action on the mucous membrane of the throat. An antiseptic solution pleasant in taste and smell and slightly astringent, can be made from a combination of the following, namely, thymol, menthol, oil of eucalyptus, benzoic acid, and oil of gaultheria, sig., liquor antisepticus; to these may be added boracic acid and sodium sulphocarbolate.

The following is a useful formula, one dose to be mixed with four ounces of warm water before use:

| | |
|---|---|
| Sodium bicarbonate ... ... ... | gr. 5 |
| Sodium biborate ... ... ... ... | gr. 5 |
| Sodium chloride ... ... ... ... | gr. 5 |
| Sodium benzoate ... ... ... ... | gr. $\frac{1}{3}$ |
| Oil of eucalyptus ... ... ... ... | m. $\frac{1}{6}$ |
| Thymol ... ... ... ... ... | gr. $\frac{1}{6}$ |
| Oil of gaultheria ... ... ... ... | m. $\frac{1}{12}$ |

The following is a useful combination:

| | | |
|---|---|---|
| ℞ | Liq. antiseptici ... ... ... ... | ℥iss. |
| | Sodium bibor. ... ... ... ... | ʒij. |
| | Glycerini. ... ... ... ... ... | ʒiij. |
| | Aq. ad. ... ... ... ... ... | ℥viii. |

To be mixed with an equal quantity of warm water before use as a gargle, spray, etc.

One of the cheapest applications that can be used for the throat is chlorine water, and this solution has a cleansing as well as an antiseptic effect. In severe cases of throat affection with much discharge the disagreeable taste is not much noticed. It can be made as follows: A drachm and a half of powdered potassium chlorate is placed in a strong twenty-ounce bottle, and two drachms of strong hydrochloric acid are added; the acid is allowed to mix well with the powder by rotating the bottle, the finger being at the same time kept on the cork. As soon as violent agitation ceases an ounce of water is added, and the bottle well shaken up; this process is repeated until sixteen ounces have been added, the bottle being well shaken after each addition of water; four ounces of syrup are finally added. This solution should be mixed with an equal part of warm water before use, or further diluted as required. Chlorine water will be found useful in cases of scarlet fever, and this treatment I have carried out in some thousands of cases with good results.

Formic aldehyde, on account of its disagreeable smell and irritant effect, has not been much used for throat affections. It is claimed for a new preparation, formamint, that these objections have been overcome by combining the antiseptic with lactose and a small quantity of pepsin hydrochloric acid. The substance is sold in lozenges, from which formaldehyde is liberated in the mouth; they are of pleasant taste and smell.

In cases where swabbing or painting are used in throat affections stronger solutions should be used than where the throat is syringed, douched, etc., such as chlorine water, or liquor antisepticus, with an equal quantity of water; solutions of carbolic acid, 1 in 40; biniodide of mercury, 1 in 500—1 in 1,000 (these latter have the disadvantage of being poisonous), or the following:

| | | |
|---|---|---|
| ℞ | Iodi ... ... ... ... ... | gr. ij. |
| | Pot. iodidi... ... ... ... ... | ʒss. |
| | Glycerine ... ... ... ... ... | ℥j. |

In diphtheria it is advisable to paint the throat with biniodide of mercury solution or other solution twice daily, even after all membrane and inflammation has disappeared, to get rid of any diphtheria bacilli present in the throat.

In mild cases, and where the inflammation is subsiding in severe and moderately severe cases, solutions less marked in their antiseptic action or those of an astringent nature can be used, *e.g.*:

1. A saturated solution of boracic acid in water. 2. Alum or potassium chlorate, gr. x. to an ounce of water. 3. The preceding in combination with tincture of myrrh, m. x.

4. Borax, gr. iij., in combination with potassium chlorate, gr. x., to an ounce of water.

The above solutions should be mixed with an equal quantity of warm water before use. The throat can also be painted with glycerine of tannic acid.

If there is much pain on swallowing, painting with a 5-10 per cent. solution of cocaine will give relief, while pain and swelling in the glands of the neck are relieved by the application of hot fomentations.

The local treatment referred to is applicable to all acute infective conditions of the throat, including syphilitic affections, membranous forms of tonsillitis other than diphtheria and simple acute tonsillitis.

